# Supplementary material for: Evidence supported by Mendelian randomization: impact on inflammatory factors in knee osteoarthritis
Source: Front Med (Lausanne). 2024 May 28;11:1382836. doi: 10.3389/fmed.2024.1382836 (PMC11165061; doi:10.3389/fmed.2024.1382836)
Supplement: Supplementary file 1 [file Data_Sheet_1.ZIP › data/GCST90274783_5e6/again/783.forest.pdf]

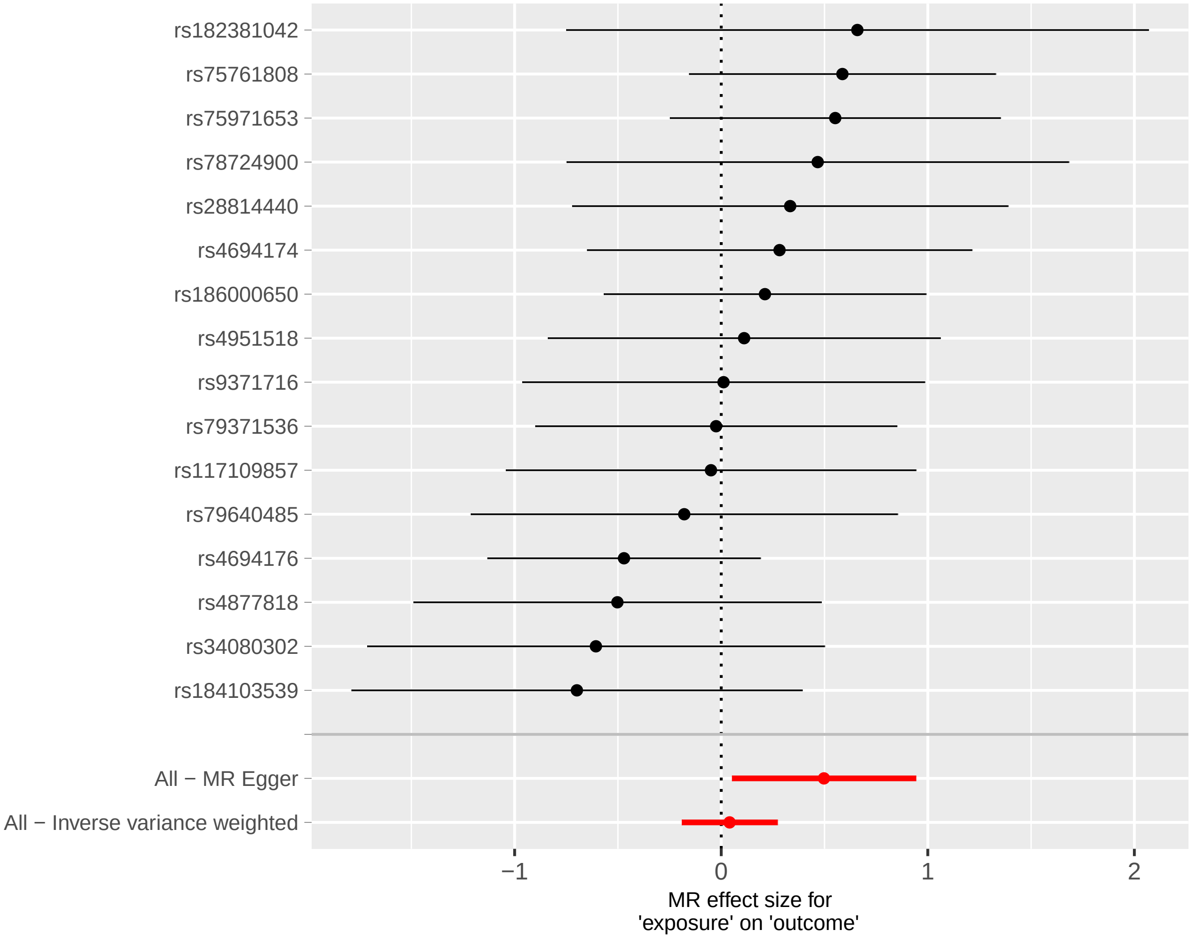
rs182381042
rs75761808
rs75971653
rs78724900
rs28814440
rs4694174
rs186000650
rs4951518
rs9371716
rs79371536
rs117109857
rs79640485
rs4694176
rs4877818
rs34080302
rs184103539
All − MR Egger
All − Inverse variance weighted
−1
0
1
2
MR effect size for
'exposure' on 'outcome'
